# Phenylboronic ester-modified anionic micelles for ROS-stimuli response in HeLa cell

**DOI:** 10.1080/10717544.2020.1748761

**Published:** 2020-05-12

**Authors:** Qi Y. Wang, Yi S. Xu, Nan X. Zhang, Zhi P. Dong, Bo N. Zhao, Lin C. Liu, Tao Lu, Yue Wang

**Affiliations:** a Key Laboratory of Biomedical Functional Materials, School of Sciences, China Pharmaceutical University, Nanjing, China;; b Department of Rheumatology, Zhongda Hospital, School of Medicine, Southeast University, Nanjing, China

**Keywords:** Anionic micelles, ROS-Stimuli response, drug delivery system

## Abstract

Smart polymers as ideal drug nanocarriers have attracted much attention due to the effective drug delivery, internalization and release once triggered by intracellular stimuli, as well as reduced cytotoxicity. We here reported the anionic micelle consisting of copolymer (PEG-b-PAsp) and a PBE (Phenylboronic Ester) group grafted, which can achieve fast response to intracellular ROS and enhanced anti-tumor activity. With this, PEG-b-PAsp-g-PBE/DOX system showed better tumor growth inhibition when studied on HeLa cell lines with high level of intracellular ROS and its subcutaneous tumor models. Additionally, the administration of PEG-b-PAsp-g-PBE/DOX did cause significantly lower systemic toxicity in comparison with free DOX. Hence, PEG-b-PAsp-g-PBE could be a highly efficient and safe nanocarrier to improve the efficacy of chemotherapeutic.

## 1. Introduction

Over the past decades, cancer has been the most common cause of death second only to heart diseases. Chemotherapy plays a vital role in inhibiting many malignant tumor growth. However, the clinical application of chemotherapeutics was limited by the extremely serious adverse effect. Therefore, various biodegradable nanocarriers (Ulbrich et al., 2016; Cabral et al., 2018; Liu et al., 2019) have been designed as drug vehicles for cancer therapy, including liposomes (Wijetunge et al., 2018; Dai et al., 2019), micelles (Sun et al., 2018; Zhang et al., 2018; Hua et al., 2019), polymersomes (Liang et al., 2018; Einfalt et al., 2018). Ideal nanocarriers should be able to efficiently protect chemotherapeutics from degradation and clearance during circulation, specifically deliver chemotherapeutics to tumor cells, and precisely control and release drug (Zhang et al., 2019). To achieve this, a variety of stimuli-responsive nanocarriers have been designed and utilized for cancer therapy, which can become cleavable and physical chemical properties in response to tumor microenvironment triggers (Timko et al., 2010; Hu et al., 2017; Lu et al., 2017; Chen et al., 2018).

ROS (Reactive Oxygen Species) and oxidative stress as an excellent triggers is associated with distinct pathological conditions, especially cancer (Li et al., 2018). Compared with normal cells, the level of ROS in cancer cells are evidenced to be 100 times higher. Therefore, the increased level of ROS provides an emerging biomaterial basis in the field of internal biological stimuli (Xu et al., 2016; Xiang et al., 2018). Recently, PBA(phenylboronic acid)-based ROS-responsive vesicles implicated in nanomedicine for cancer targeting have aroused scientific interest because of an important superiority of high drug loading capacity, fast responsiveness, and excellent biocompatibility. A pioneering work by Liu was to construct the PBA contained-polymersome for intracellular delivery nanocarriers and nanoreactors to enhance imaging/drug release features. Upon cellular uptake, PBE group can be selectively degraded by intracellular H_2_O_2_ triggers, generating phenol and boronic acid as the oxidation products (Xu et al., 2016).

Herein, we reported the fabrication of ROS-responsive micelles exhibiting intracellular triggered and enhanced cancer-targeting features (Scheme 2). During optimization of the chemical design in terms of loading capacity and biocompatibility, we mainly synthesized the block copolymers of polyethylene glycol (PEG)-poly aspartic acid (m-PEG-b-PAsp) with proper PAsp chain lengths. The micelles are self-assembled from block copolymer incorporated with PEG and phenylboronic ester (PBE)-conjugated PAsp (PEG-b-PAsp-g-PBE) and have DOX encapsulated through electrostatic interaction in the interior. The copolymer PEG-b-PAsp-g-PBE loses its PBE side chains and becomes water soluble, leading to release of the preloaded DOX under the high level of ROS in HeLa cells. They hold great promise for controlled drug delivery due to its large loading capacity of DOX and fast responsiveness. Afterwards the drug efficiency of micelles was investigated both *in vitro* and *in vivo*. It is anticipated that the micelles can be utilized for cancer-target delivery with using chemically modified PBE as an ROS-degradable and biocompatible polypeptides material.

## 2. Results and discussion

### 2.1. Synthesis of diblock copolymer PEG-b-PAsp-g-PBE

**Scheme 1.**
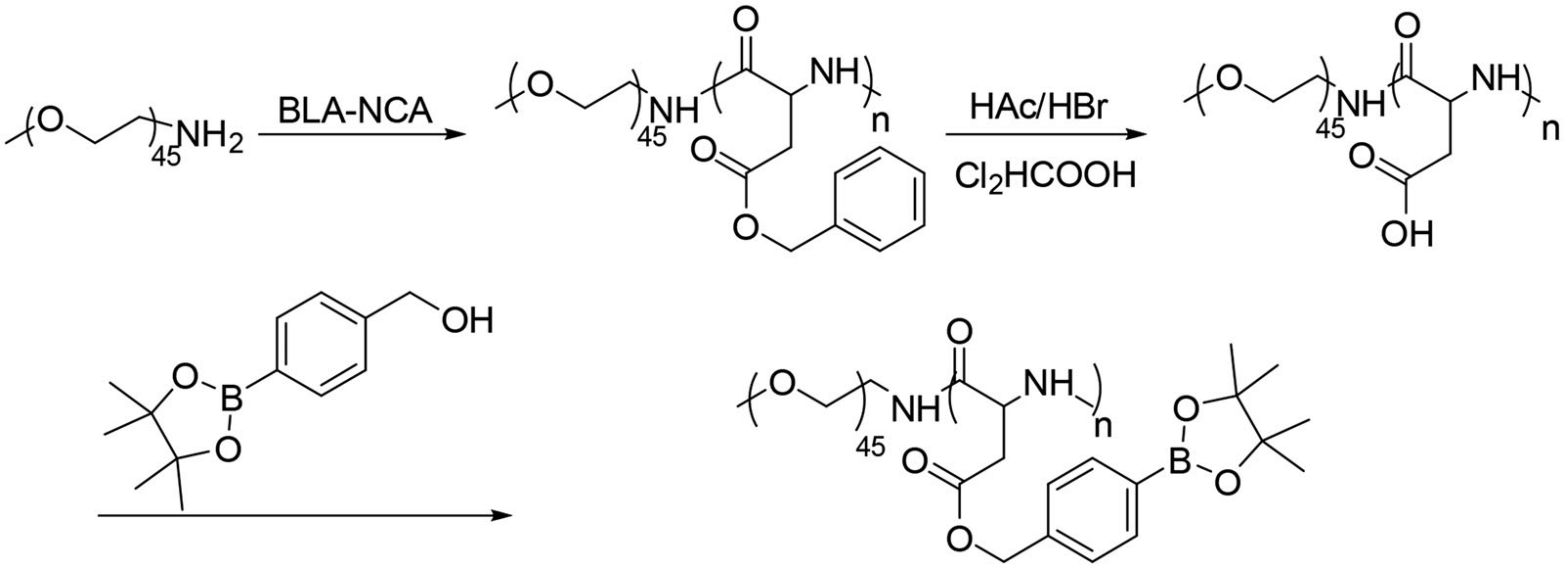
Synthesis of PEG-b-PAsp-g-PBE Copolymers.

The PEG-b-PAsp-g-PBE copolymer was synthesized through two steps as shown in Scheme 1. The first step was to prepare copolymer PEG-b-PAsp. Copolymers PEG-b-PBLA were synthesized via the amine-initiated ring-opening polymerization (ROP). The amphiphilic PEG-b-PAsp diblock copolymers were obtained after the deprotection of PEG- b-PBLA by HBr/HAc in dichloroacetic acid solution. Finally, according to the length of PAsp chain, PEG-b-PAsp-g-PBE was performed through esterification reaction, conjunction of the phenylboronic acid into the PAsp chain.

**Scheme 2.**
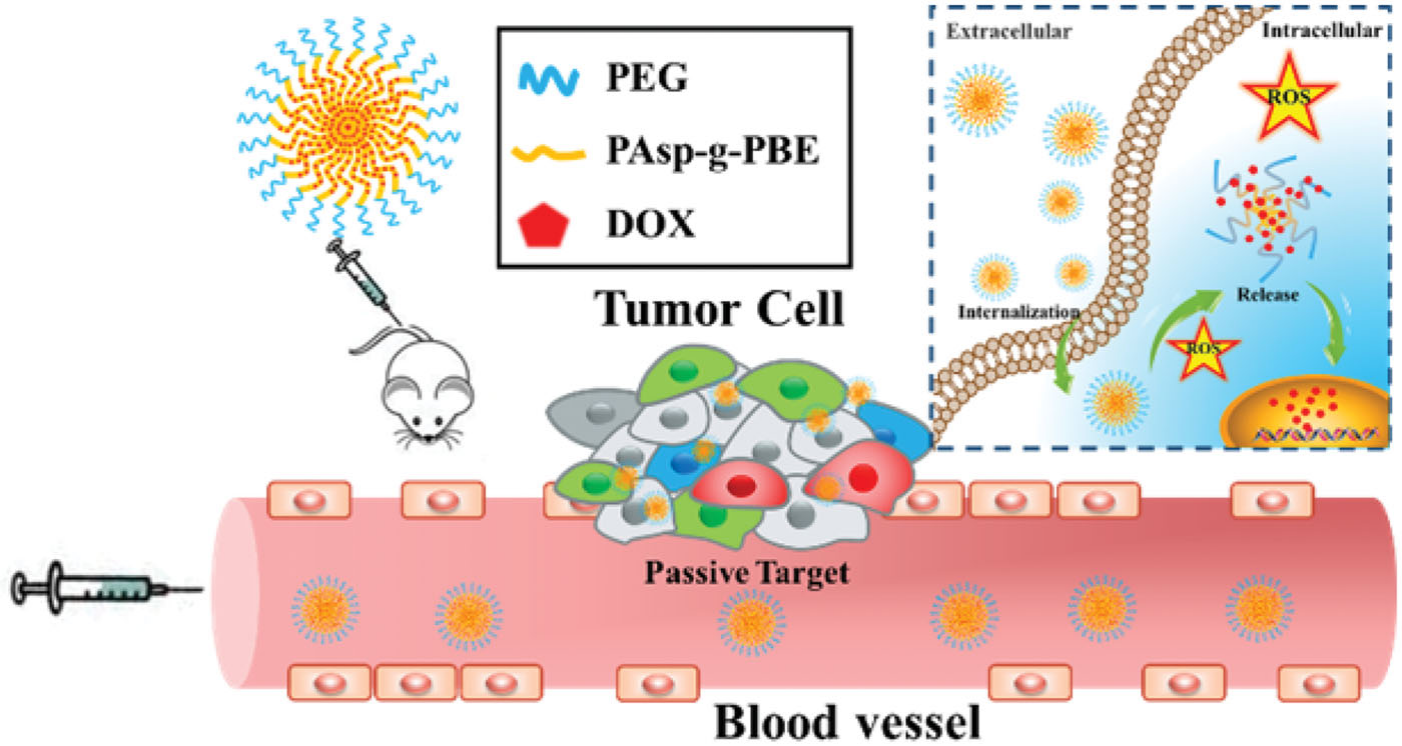
Schematic illustration of PEG-b-PAsp-g-PBE/DOX as an antitumor drug delivery system.

**Figure 1.**
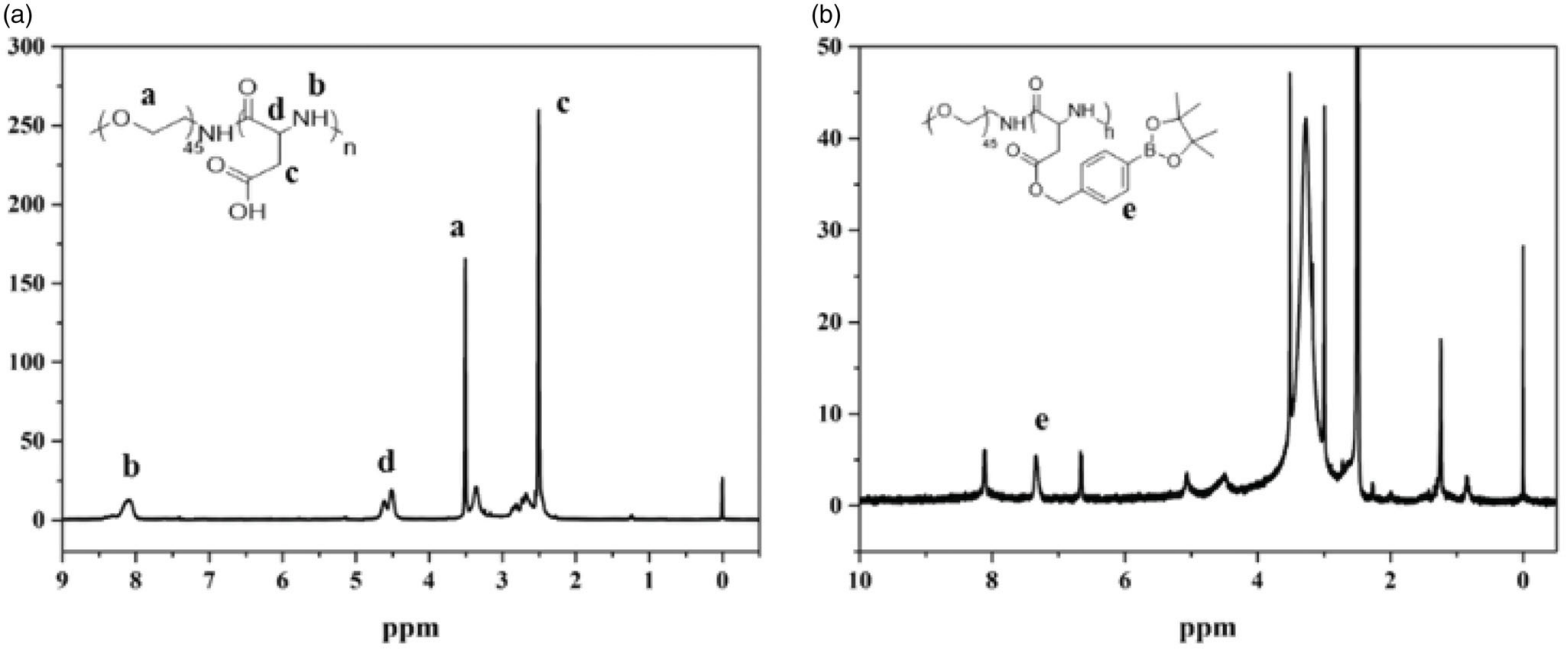
(a) ^1^H NMR spectra of PEG-b-PAsp in DMSO (b) ^1^H NMR spectra of PEG-b-PAsp-g-PBE in D_2_O.

**Figure 2.**
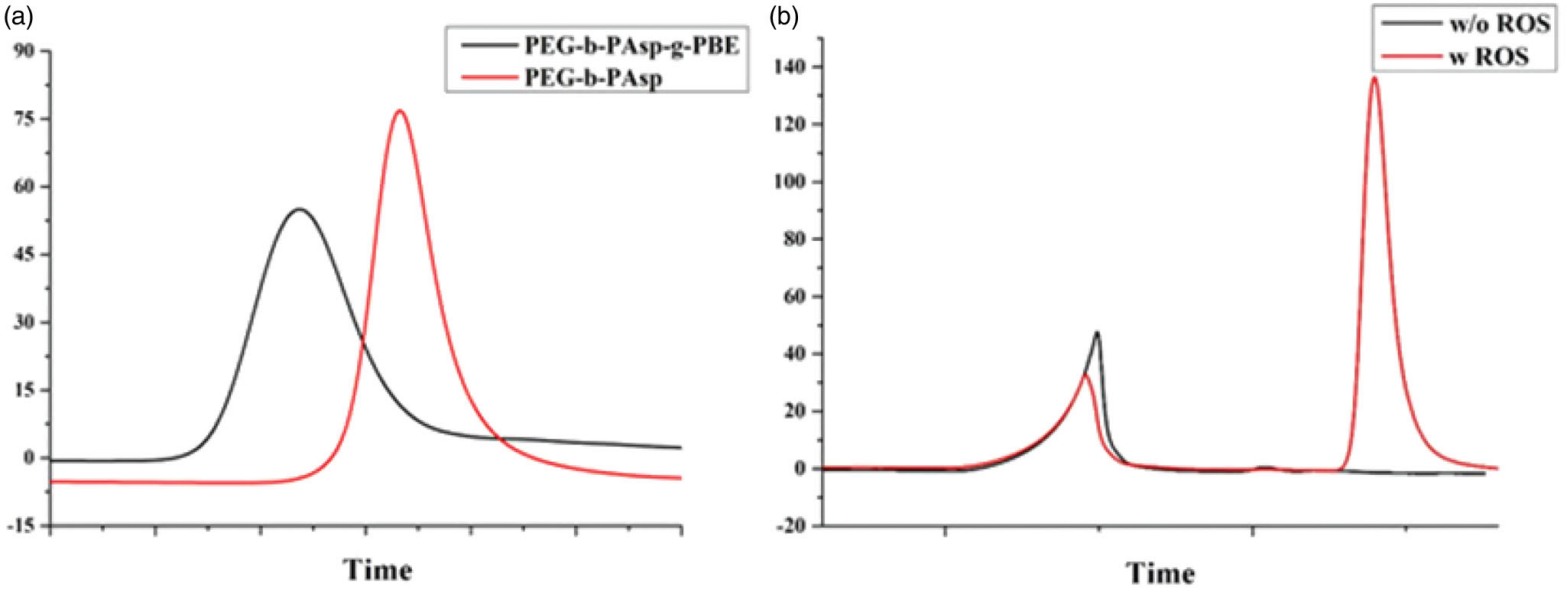
Gel permeation chromatography (GPC) of copolymers (a) PEG-b-PAsp and PEG-b-PAsp-g-PBE (b) PEG-b-PAsp-g-PBE treated with ROS.

The structure of the copolymers (PEG-b-PAsp and PEG-b-PAsp-g-PBE) was characterized using ^1^H-NMR spectroscopy. As shown in Figure 1(a), the peaks around 3.51(a) ppm are attributable to the protons (-CH_2_CH_2_O) in the PEG chain. The peak around 8.06(b) ppm, 4.5(c) ppm and 2.7(d) ppm are attributable to the protons (-CONH-, -COCHNH-, CH_2_COOH) in the PAsp chain. The polymerization degrees of PAsp and PEG were determined by calculating the ratio of peak areas of 3.51 ppm to 8.06 ppm, respectively. In Figure 1(b), the peaks around 6.8 ppm, 7.4 ppm and 8.1 ppm (e) attributable to the protons of phenylboronic acid. The degree of PAsp grafted by PBE was calculated by the ratio of peak areas of the protons in the PEG chain and peak of the aromatic protons in the PBE phenyl rings. GPC traces of the block copolymers, PEG_45_-b-PAsp_72_ and PEG_45_-b-PAsp_72_-g-PBE_41_ in Figure 2(a) are invariably unimodal with little or no tailing. The compositions of PEG-b-PAsp and PEG-b-PAsp-g-PBE diblock polymers were listed in Table 1. After 4 hours, the GPC result showed one more distinct peak appeared with addition of ROS at pH 7.4, indicating PBA-grafted polymer can be degraded in response to H_2_O_2_ triggers (Figure 2(b)).

**Table 1.** Feed Composition and Final Composition of PEG-b-PAsp as well as the CMC Value of the Polymer.

Diblock Polypeptide	Mn^a^ 10^3^	PDI^b^	CMC
PEG_45_-b-PAsp_72_	10.0	1.53	5.6 mg/L
PEG_45_-b-PAsp_72_-g-PBE_41_	20.0	1.50	2.8 mg/L

a Calculated from ^1^H NMR analysis, the number-average molecular weight (Mn).

b Calculated from GPC analysis, the polydispersity index (PDI).

### 2.2. *In vitro* cytotoxicity of copolymer

**Figure 3.**
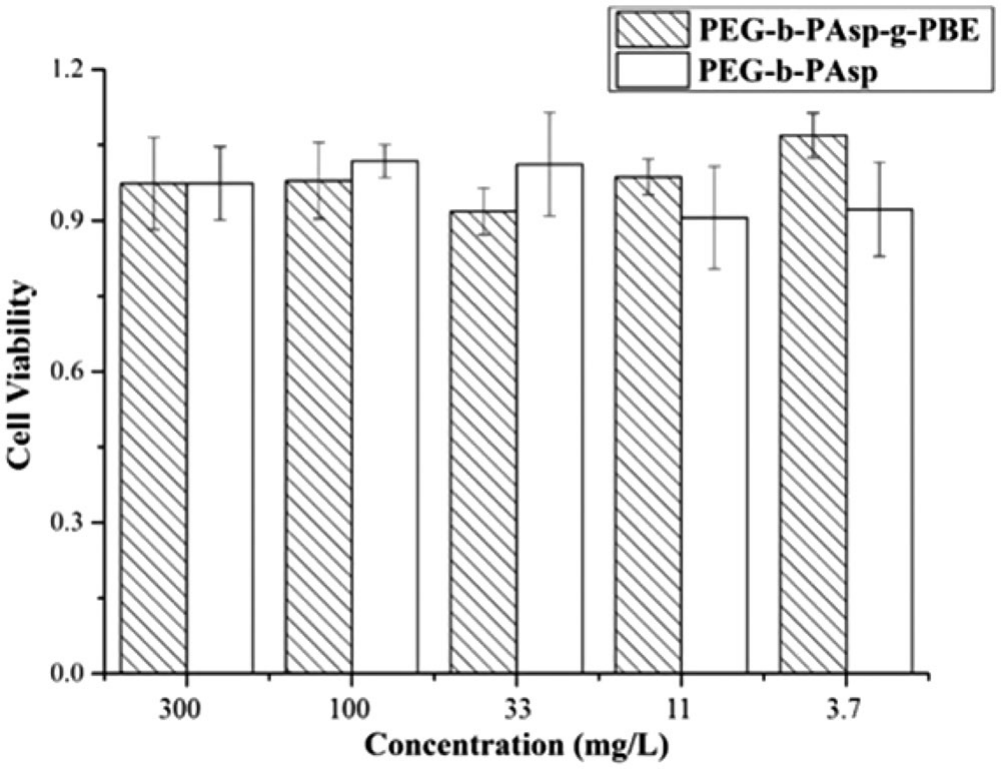
Cell inhibition of PEG-b-PAsp and PEG-b-PAsp-g-PBE on HeLa at different concentrations.

Cytotoxicity of the different copolymer was respectively evaluated by MTT assay and HeLa cells were used in this study. As shown in Figure 3, 3.7–300 mg/l (3.7, 11, 33, 100, 300) of PEG-b-PAsp and PEG-b-PAsp-g-PBE copolymers did not significantly affect the viability of HeLa cell after 48 h of incubation. Consistent with HeLa cell line, the results showed that there was a dramatically high cell viability (more than 95% even at the highest concentration of 300 mg/l) in normal cell line L-O2 (Figures S1 and S2), which displays low cytotoxicity. Inspired by its low cytotoxicity, we finally chose PEG_45_-b-PAsp_72_ and PEG_45_-b-PAsp_72_-g-PBE_41_ as the basic diblock copolymer to carry out the further experiments.

### 2.3. Preparation of polymeric micelles and drug release *in vitro*

The PEG-b-PAsp-g-PBE and PEG-b-PAsp-g-PBE/DOX micelles were prepared by dialysis method. The morphology was characterized transmission electron microscope (TEM) and dynamic light scatting (DLS).

**Figure 4.**
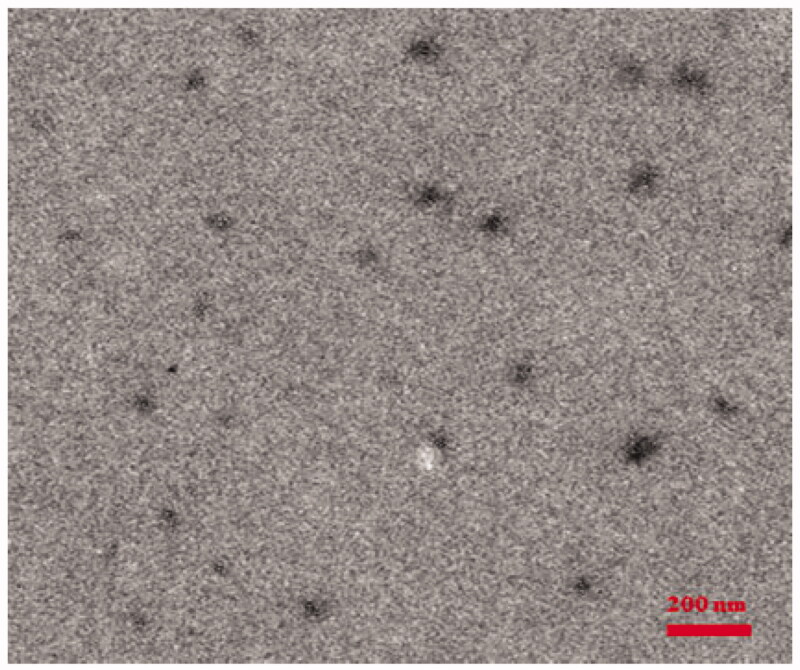
TEM image of the PEG_45_-b-PAsp_72_-g-PBE_41_/DOX.

The hydrodynamic diameter of the PEG-b-PAsp micelle was determined as about 70 nm by TEM (Figure S3) which is consistent with its hydrodynamic diameter (Figure S4). Compared with PEG-b-PAsp, its size increased with the modification of PBE group (Figure S5). Moreover, the zeta potential of PEG-b-PAsp-g-PBE micelles changed from −23.9 mV to −15.7 mV (Figure S6), which can further confirm the PBE group has been modified successfully. However, the average diameter of PEG-b-PAsp-g-PBE/DOX was determined as 95 nm by TEM (Figure 4) and DLS (Figure S7), which increase with the effect of the dox encapsulation. Their, surface charges vary from −23.9 mV to −16.1 mV, demonstrating that DOX has been loaded through the electrostatic interaction (Rejinold et al., 2018).

**Figure 5.**
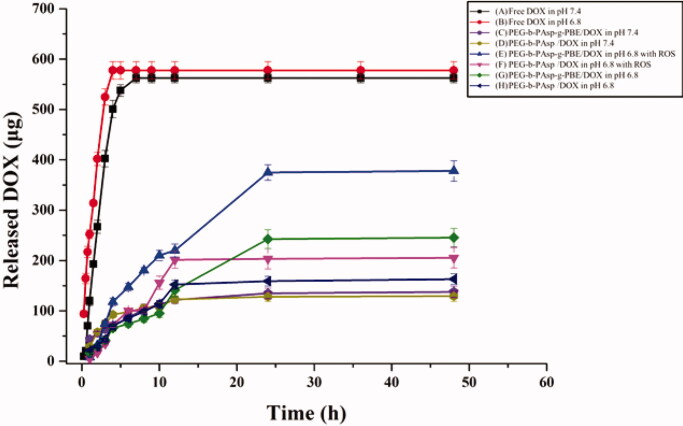
*In vitro* release profiles of DOX.

We next assessed the *in vitro* DOX release profile of the micelles (PEG-b-PAsp/DOX, DLE = 42.0%, DLC = 26.1% and PEG-b-PAsp-g-PBE/DOX, DLE = 43.2%, DLC = 30.0%) in response to ROS by incubating the micelles with PBS, including a control level (pH = 7.4), an acidic pH level (pH = 6.8) and an acidic pH level with ROS (pH = 6.8 with 1 mM H_2_O_2_). Free DOX was released quickly in 3 hours at pH 6.8. Even at pH 7.4, it released totally within 6 hours. Since DOX has good water solubility, the free DOX can rapidly exchanges in blood. Compared with the burst release of free DOX, the PEG-b-PAsp/DOX and PEG-b-PAsp-g-PBE/DOX exhibits a sustained release process and the controlled release behavior. As shown in Figure 5, the release of DOX from PEG-b-PAsp/DOX and PEG-b-PAsp-g-PBE/DOX was low (22% and 30%) at normal physiological pH 7.4 after 48-h incubation. Acidification of the buffer to pH 6.8 caused a moderately higher release rate (37% and 45%), which could confirm proton can break electrostatic interaction between amino group from DOX and carboxyl group from PAsp, causing drug releasing. Moreover, a significantly higher release rate (70%) of DOX from PEG-b-PAsp-g-PBE/DOX was observed at acidic pH level with H_2_O_2_ addition, while DOX release from PEG-b-PAsp/DOX did not obviously increase under the same condition. Therefore, PBA group can be degraded by H_2_O_2_ triggers and causing the micelle collapse. Compared with PEG-b-PAsp/DOX, PEG-b-PAsp-g-PBE/DOX exhibited not only a typical pH-dependent, but also ROS-dependent manner due to the grafted PBE molecules.

### 2.4. Intracellular ROS generation levels in different cell lines

**Figure 6.**
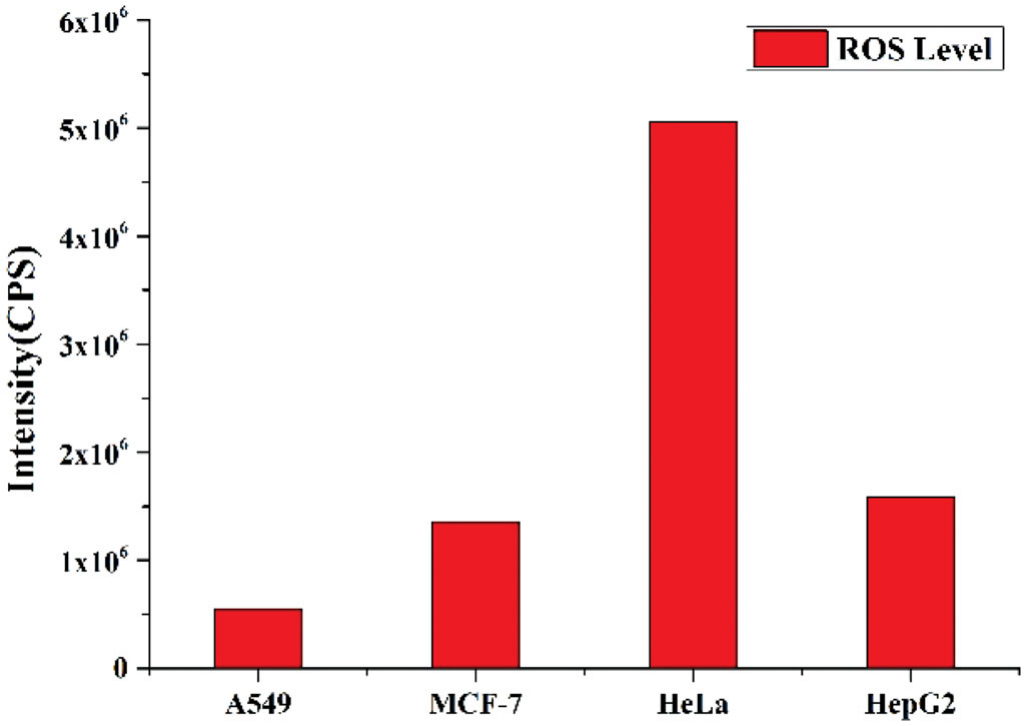
ROS levels in A549, MCF-7, HeLa and HepG2 cells quantified using DCFH-DA reagent by fluorometric analysis.

Encouraged by the good ROS-dependent manner of PEG-b-PAsp-g-PBE/DOX, we investigated intracellular ROS levels spontaneously generated in different cell lines (A549, MCF-7, HeLa, and HepG2 cells) by using a ROS Assay Kit (Luan et al., 2019). DCFH-DA, a fluorescence probe for ROS detection, was employed to evaluate the level of intracellular ROS. As depicted, the intracellular ROS levels of HeLa cells were significantly higher in comparison with MCF-7, HepG2 and A549 cells (Figure 6). Encouraged by its level of intracellular ROS, we chose HeLa cell to perform the following experiments.

### 2.5. *In vitro* cellular internalization and cytotoxicity of PEG-b-PAsp/DOX, PEG-b-PAsp-g-PBE/DOX micelles

**Figure 7.**
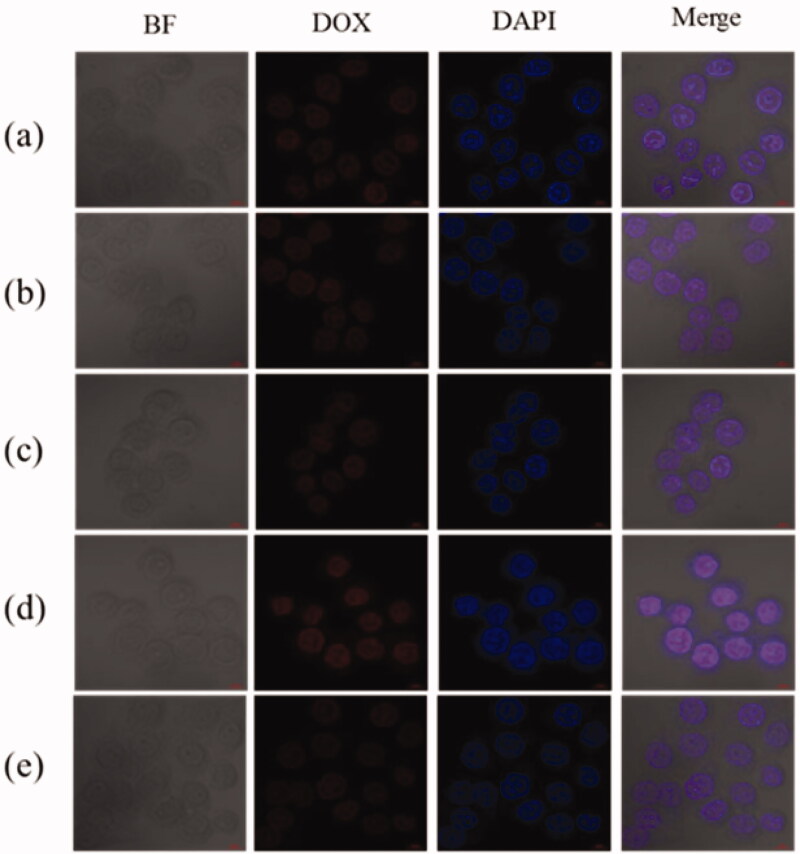
Cellular uptake and intracellular localization of DOX in HeLa cells. (a) Free DOX (b) PEG-b-PAsp/DOX (c) PEG-b-PAsp/DOX with NAC pretreatment (d) PEG-b-PAsp-g-PBE/DOX (e) PEG-b-PAsp-g-PBE/DOX with NAC pretreatment.

The effect of PBE functionalization on cellular uptake was evaluated by HeLa cells by confocal laser microscopy. For DOX itself is fluorescent, it was used directly to investigate cellular uptake without additional markers in the micelles. Figure 7 showed the cell uptake and intracellular distribution of PEG-b-PAsp/DOX, PEG-b-PAsp-g-PBE/DOX micelles in HeLa cells after incubation for 4 h. HeLa cells treated with PEG-b-PAsp-g-PBE/DOX (10 μg/mL) presented significantly higher fluorescent signal (Figure 7(d)), indicating a more cellular uptake than that of free DOX (Figure 7(a)) and PEG-b-PAsp/DOX (Figure 7(b)) in the nuclei. Further to prove the role of PBA molecule in ROS response, we pretreat the cells with NAC, acting as a scavenger of ROS, which led to a remarkable decrease of the cellular internalization of PEG-b-PAsp-g-PBE/DOX (Figure 7(e)). However, in PEG-b-PAsp/DOX group (Figure 7(c)), fluorescent signal of DOX did not decrease in pretreatment cell. PEG-b-PAsp-g-PBE effectively facilitated the uptake of DOX through the fast response to the high level of intracellular ROS on HeLa cell.

Besides, DOX released from the PEG-b-PAsp-g-PBE/DOX were quickly transported to the cytoplasm and diffused to the nuclei in response to high concentration of ROS and inherent intracellular acid. Then, the diffused DOX specifically affiliated with DNA and lead to tumor cells damage and apoptosis. As shown in bright field in Figure 7, most HeLa cells treated with the free DOX still survived, while most of cells died with the PEG-b-PAsp-g-PBE/DOX treatment. It indicated that the PEG-b-PAsp-g-PBE/DOX showed the more efficient cell inhibition dependent on the concentration and presented notable cytotoxicity toward HeLa cells, as evidenced with MTT assays.

**Figure 8.**
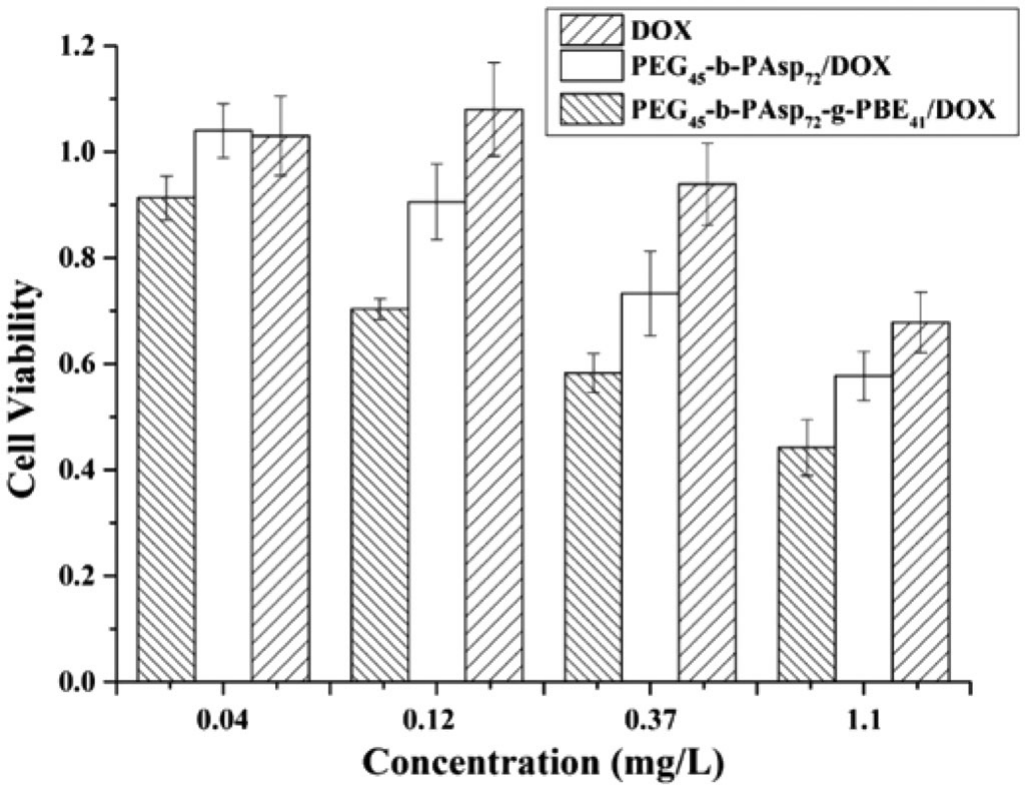
Cell viability rate of HeLa cell lines after co-incubation with DOX, PEG_45_-b-PAsp_72_/DOX and PEG_45_-b-PAsp_72_-g-PBE_41_/DOX for 48 h, respectively.

The effect of PBE modification on the cytotoxicity of drug-loaded micelles was studied with the cancer cells HeLa. After 48-h incubation, dose-dependent loss of cell viability was reduced in free DOX, PEG-b-PAsp/DOX, PEG-b-PAsp-g-PBE/DOX group (Figure 8). This confirms that the cytotoxicity was due to delivery of the DOX but not the micelles itself. Free DOX, PEG-b-PAsp/DOX, PEG-b-PAsp-g-PBE/DOX all exhibited dose-dependent cytotoxicity on HeLa cells. The cytotoxicity of PEG-b-PAsp-g-PBE/DOX was superior to free DOX and PEG-b-PAsp/DOX in cell lines HeLa. Even at the low concentration (0.12 mg/L), PEG-b-PAsp-g-PBE/DOX displays the obvious inhibition, while free DOX and PEG-b-PAsp/DOX does not. The micelles with ROS trigger exhibited an efficient inhibition of HeLa cell growth than that without ROS trigger and free DOX, which is consistent with CLSM result.

### 2.6. *In vivo* tumor inhibition of PEG-b-PAsp-g-PBE/DOX

Encouraged by *in vitro* effective antitumor activity and uptake on HeLa cells, we proceeded to test the antitumor efficacy of PEG-b-PAsp-g-PBE/DOX in HeLa cell inoculated xenograft mice. Mice were used for the *in vivo* tumor inhibition experiments, as the tumor volume reached about 120–150 mm^3^.

We divided tumor-bearing mice into five different groups and later intravenously injected into mice with PEG-b-PAsp-g-PBE/DOX from tail vein. Two group treated with PBS and PEG-b-PAsp-g-PBE were recruited as blank group and another two groups treated with DOX and PEG-b-PAsp/DOX positive controls for 18 days. In order to investigate the tumor growth after administration, we periodically measured the tumor sizes after tail injection.

**Figure 9.**
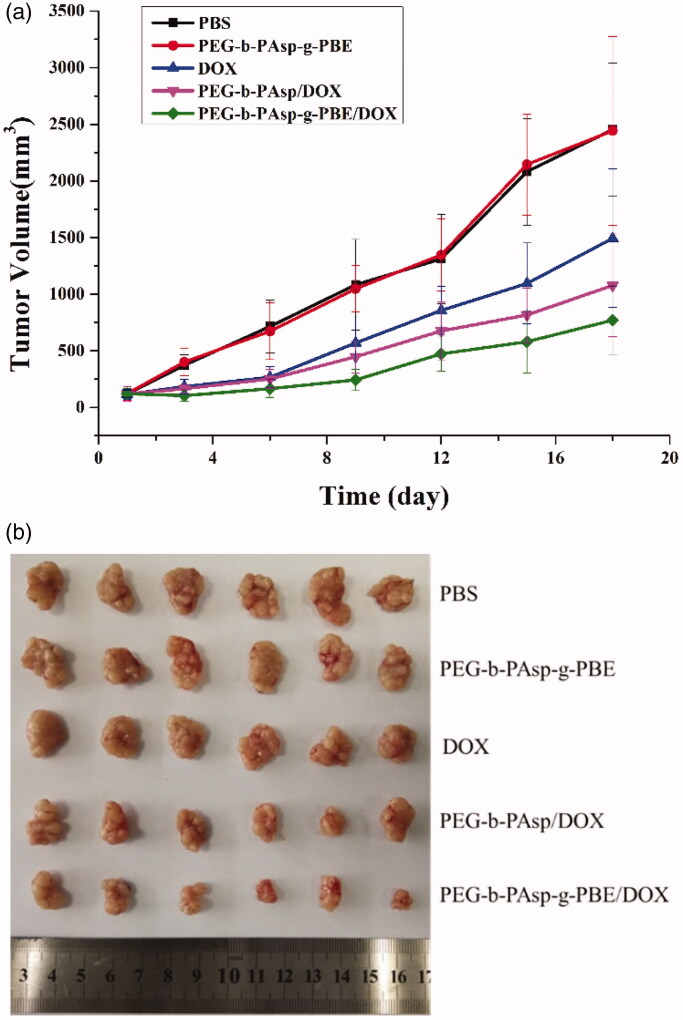
(a) Real-time observation of tumor sizes in vivo after treatment with samples. Error bars represent means ± SD (*p <.05, **p <.01); (b) Photo of tumor tissues from mice treated with PBS, PEG-b-PAsp-g-PBE, DOX, PEG-b-PAsp/DOX, PEG-b-PAsp-g-PBE/DOX at the end of the study.

After injection with blank samples (PBS saline and PEG-b-PAsp-g-PBE), no inhibitory effect on tumor growth was observed, and the average tumor volumes of the two control groups at the end of the experiments were 2452.09 and 2442.70 mm^3^, respectively. However, all DOX groups (DOX, PEG-b-PAsp/DOX and PEG-b-PAsp-g-PBE/DOX) showed different levels of tumor growth inhibition effect. The tumor volume and tumor growth inhibition of the group PEG-b-PAsp-g-PBE/DOX injected with was 770 mm^3^ and 68.9% on the 18th day, while positive group treated with DOX and PEG-b-PAsp/DOX were 1492.9 mm^3^, 39% and 1078.4 mm^3^, 58.05%, respectively. The holistic tumor photograph after excision was visible in Figure 9. Compared with the saline group, the group treated with PEG-b-PAsp/DOX, PEG-b-PAsp-g-PBE/DOX and DOX showed smaller tumor burden. Remarkable inhibition in tumor growth was observed with PEG-b-PAsp-g-PBE/DOX during the in vivo study in comparison to free DOX and PEG-b-PAsp/DOX. At the same concentration (2 mg/kg) of DOX, experiments in mice demonstrated that PEG-b-PAsp-g-PBE/DOX did have more efficient antitumor activity *in vivo* than free DOX which is used as a first-line chemotherapy drug against HeLa tumor. This result can demonstrate PBE groups take advantage of passive targeting to deliver DOX to malignant tumor more efficiently.

**Figure 10.**
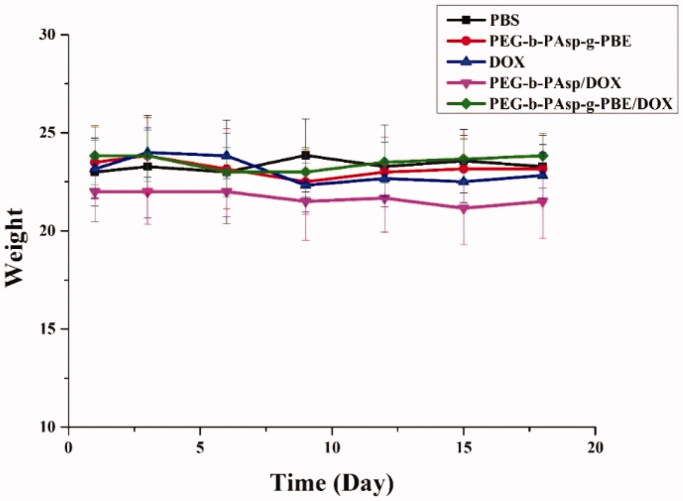
Real-time weight analysis of mice after each treatment. Error bars represent means ± SD.

Toxicity is a major concern for *in vivo* application of chemotherapy. In order to identify the safety of drug delivery, we also measured the changes of mouse weight during the treatment process. We discovered that the weight of all five groups of mice did not change obviously at the end of treatment. (Figure 10) Meanwhile after treatment with PBS, PEG-b-PAsp-g-PBE, free DOX, PEG-b-PAsp/DOX and PEG-b-PAsp-g-PBE/DOX for 18 days, we sacrificed mice, collected all the tumor tissues and measured them respectively (Liu et al., 2016; Wang et al., 2019).

**Figure 11.**
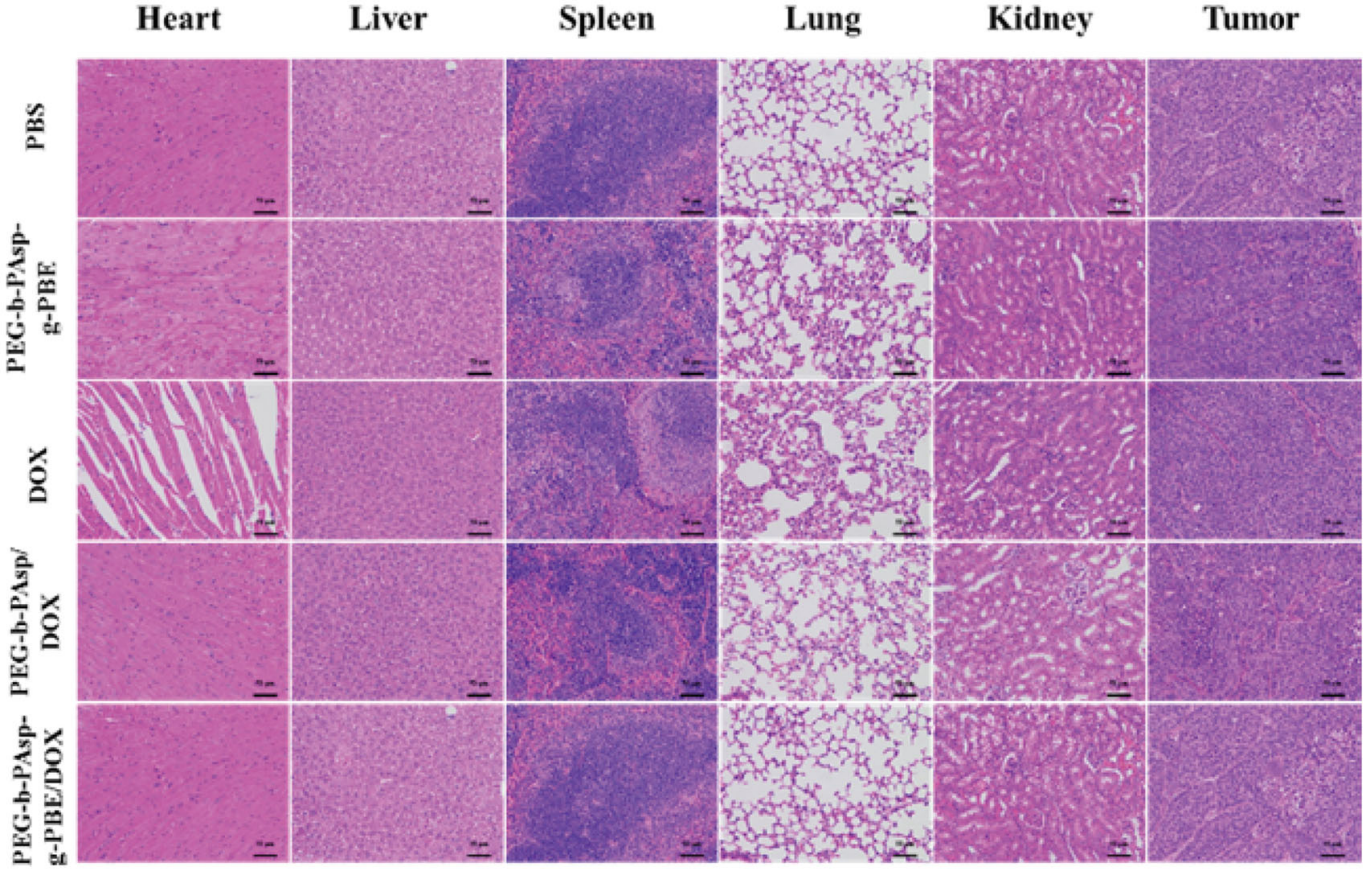
Histological analysis of tissues with H&E after treatments with PBS, PEG-b-PAsp-g-PBE, DOX, PEG-b-PAsp/DOX, PEG-b-PAsp-g-PBE/DOX.

The histological analysis of fixed tissues was showed in Figure 11. DOX·HCl, a classical chemotherapeutic agent, is well known for its severe side effects dominated by heart toxicity. Compared with tissues of blank group, especially in histological observation of heart tissues, puff cardiomyocyte nucleus with vacuole and undense cytoplasm, was observed in free DOX group. While in PEG-b-PAsp-g-PBE/DOX group, no obvious abnormality was found. Obviously, through the reasonable design and the chemical assembly, the damages on heart due to the low selectivity of DOX·HCl were efficaciously relieved by this intelligent drug delivery system.

## 3. Conclusion

In summary, we have constructed the biocompatible drug delivery system with a polypeptide material PEG-b-PAsp-g-PBE, containing PBE chemically modification for effective chemotherapeutic drugs delivery. In comparison with PEG-b-PAsp/DOX, the higher inhibition of fabricated ROS-responsive PEG-b-PAsp-g-PBE/DOX is mainly owing to valid degradation, efficient disassembly and successful intracellular release of DOX once triggered by intracellular ROS. PBE groups can enhanced their tumor targeting ability passively by fast response to tumor microenvironment of ROS rich. Benefiting from the PBE, the entire system showed an enhanced chemotherapeutic efficacy both *in vitro* and *in vivo*. Overall, PEG-b-PAsp-g-PBE exhibited a good tumor inhibition effect and low side effects, which will open a new avenue toward the fabrication of smart drug delivery system by taking advantage of biologically relevant intracellular triggering stimuli.

## 4. Experiment section

### 4.1. Chemicals and apparatus

All reagents and solvents were commercially available and used without additional treatment. Doxorubicin Hydrochloride (DOX•HCl, denoted as DOX, 99.8%) were purchased from Melone Pharmaceutical Corporation. Dulbecco’s modified Eagle’s medium (DMEM), fetal bovine serum (FBS), 3-(4,5-dimethylthiazol-2-yl)-2,5-diphenyltetrazolium bromide (MTT), trypsin-EDTA, penicillin–streptomycin, dimethyl sulfoxide (DMSO), and 4,6-diamidino-2-phenylindole (DAPI) were obtained from Gibco. 96 well plates, 6-well plates, and 10 mL graduated sterile centrifuge tubes were purchased from KeyGen BioTECH. Other reagents and chemicals were at least analytical reagent grade.

### 4.2. Characterization

^1^H NMR spectra of the polymers were recorded on a Bruker 400 MHz nuclear magnetic resonance instrument using DMSO as the solvents. Gel permeation chromatography (GPC) was used to analyze the molecular weights and molecular weight distributions (Mw/Mn) of the polymers. GPC of PEG-b-PAsp was measured at room temperature with a Waters 1525 chromatograph equipped with a Waters 2414 refractive index detector. H_2_O was used as eluents with a flow rate of 1.0 mL/min and narrowly distributed polyethylene glycol was used as standard. The size and surface charge of the nanocarrier was investigated on Malvern Zetasizer Nano ZS 90 zeta potential analyzer. Ultraviolet-visible (UV-vis) spectra were collected using a LAMBDA-35 spectrometer. Transmission electron microscopy (TEM) was performed on a JEOL-2100 with accelerating voltage of 200 kV. TEM samples were prepared by drop-casting dispersion onto copper grids covered by carbon film. Confocal images were acquired using a Zeiss confocal laser scanning unit mounted on an LSM 710 fixed-stage upright microscope (CLSM).

### 4.3. Synthesis of diblock copolymers

#### 4.3.1. M-poly (ethylene glycol)-b- poly (aspartate)

The diblock copolymer (PEG-b-polyaspartate) was firstly synthesized via the amine-initiated ring-opening polymerization (ROP) of N-carboxy-α-amino acid anhydrides of β-Benzyl-L-aspartate (BLA-NCA). Briefly, BLA-NCA was dissolved completely in DMF/DCM followed by addition of initiator which had been dissolved in DCM. Then, the reaction mixture was stirred for 5 days at 35 °C under a dry nitrogen atmosphere and the crude products were precipitated in 10-fold excess of cold diethyl ether and isolated by centrifugation. After washed twice with diethyl ether, the products were dried in vacuum. Block copolymers of m-PEG-b-PAsp were obtained by deprotection of m-PEG-b-PBLA in HCCl_2_COOH/HBr/HAc solution. After stirred for 3 h under ice bath, the solution was precipitated in large amount of cold diethyl ether and isolated by centrifugation. The solid was dissolved in DMSO. The solution was dialyzed against water in a dialysis bag with a proper molecular cutoff and then solid m-PEG-b-PAsp was obtained by lyophilization (Yang et al., 2015; Yavvari et al., 2019).

#### 4.3.2. M-poly (ethylene glycol)-b-poly (aspartate)-g-phenylboronic acid pinacol ester

Partial modification of PEG-b-PAsp with phenylboronic acid pinacol ester (PBE) was prepared according to the esterification reaction. First, PEG-b-PAsp was dissolved in anhydrous DMF at room temperature. According to the length of PAsp chain, PBE with proper molar ratio was added followed by DMAP addition, and the mixture solution was stirred at 40 °C for 12 h followed by being dialyzed against deionized water for 2 days in a dialysis bag with 3500DA cutoff. Finally, PEG-b-PAsp-g-PBE were obtained by lyophilizing the corresponding polymer solution (Hu et al., 2017). ^1^H NMR spectra of the polymers were recorded on a Bruker 400 MHz nuclear magnetic resonance instrument using DMSO as the solvents. Gel permeation chromatography (GPC) was used to analyze the molecular weights and molecular weight distributions (Mw/Mn) of the polymers. GPC of PEG-b-PAsp was measured by a Waters 1525 chromatograph equipped with a Waters 2414 refractive index detector. The critical micelle concentration (CMC) of PEG-b-PAsp copolymers was determined according to the literature using pyrene as a hydrophobic fluorescent probe (Raimbault et al., 2018).

### 4.4. Cell lines and culture conditions

HeLa (Cervical cancer cells), L-O2 (Human normal liver cells) A549 (Human lung adenocarcinoma cells) MCF-7 (Human breast cancer cell)and HepG2 (liver hepatocellular carcinoma) cell lines were provided by KeyGEN Biotech and maintained in Dulbecco’s Modified Eagles Medium (DMEM) containing 10% fetal bovine serum (HyClone Laboratories, Inc. Logan, UT, USA) with 100 units mL^−1^ penicillin, and 100 μg mL^−1^ streptomycin. The cells were cultured in a humidified incubator at 37 °C, 5% CO_2_. Female Balb/c mice weighing 18–20 g furnished by Experimental Animal Center, Jiangsu Academy of Traditional Chinese Medicine. All animal procedures were performed in accordance with the Guidelines for Care and Use of Laboratory Animals of China Pharmaceutical University and approved by the Animal Ethics Committee of China Pharmaceutical University, Jiangsu, China.

### 4.5. *In vitro* cytotoxicity of copolymers

The cytotoxic effect of block copolymers PEG-b-PAsp and PEG-b-PAsp-g-PBE was evaluated using MTT. In brief, L-O2 and HeLa cells were initially seeded into a 96-well cell culture plate at 4 × 103 per well and then incubated for 24 h at 37 °C under 5% CO_2_. Then, DMEM solutions with 10% FBS of different block copolymers at different concentrations were added under the same condition for 72-h incubation. Four hours before the experiment was stopped, the cells were washed three times with 0.2 mL PBS and culture medium was replaced with MTT solution of 0.2 mL. At the end of the experiment, the medium solution was replaced by 0.15 mL DMSO solution. The optical density of the solution was measured by enzyme linked immunosorbent assay (ELISA) at a wavelength of 490 nm. The absorbance value of untreated cells was set at 100%. Each experiment was repeated three times in sextuplicate. The cell viability was calculated according to following formula (Hao et al., 2018):

$$\text{the viability }(\%)=({\mathrm{OD}}_{\exp}-{\mathrm{OD}}_{\mathrm{blank}})/({\mathrm{OD}}_{\mathrm{control}}-{\mathrm{OD}}_{\mathrm{blank}})\times 100\%.$$

### 4.6. Preparation of polymeric micelles

The PEG-b-PAsp, PEG-b-PAsp-g-PBE and PEG-b-PAsp/DOX, PEG-b-PAsp-g-PBE/DOX polymeric micelles were prepared by dialysis method (Li et al., 2015).

PEG-b-PAsp (50 mg) and PEG-b-PAsp-g-PBE (50 mg) were dissolved in 4 ml DMF. Then deionized water (10 ml) was added into DMF solution slowly. The above solution was stirred at room temperature for 6 h. The mixture was dialyzed against deionized water for 3 days by using a dialysis bag (MWCO: 3500 Da). The solution in the dialysis bag was lyophilized to obtain micelles.

DOX (20 mg) and PEG-b-PAsp-g-PBE (50 mg) were dissolved in DMSO (4 ml). Then deionized water (15 ml) was added into DMSO. The above solution was stirred at room temperature for 6 h. The mixture was dialyzed against deionized water to remove DMSO and free DOX·HCl. The solution in the dialysis bag was lyophilized to obtain PEG-b-PAsp-g-PBE/DOX micelles. PEG-b-PAsp/DOX was prepared by the same method.

Drug-loading content (DLC) and drug-loading efficiency (DLE) were calculated according to the following formula:

DLC(%)=(weight of loaded drug/weight of NTs)×100%

$$\mathrm{DLE}\left(\%\right)=\left(\frac{\text{weight of loaded }\mathrm{drug}}{\mathrm{weight}\text{ of drug in feed}}\right)\times 100\%$$

Li et al., 2018.

The UV-visible absorption spectra of free DOX, PEG-b-PAsp/DOX and PEG-b-PAsp-g-PBE/DOX were scanned at 492 nm against the corresponding solvent blank in a 200 μL quartz cuvette and the calibration curve drug loading was measured in triplicate.

### 4.7. Dynamic light scattering (DLS) measurement and morphology

The size and zeta potential of polymeric micelles were measured by dynamic light scattering (DLS) using a Malvern ZS90 instrument equipped with a 532 nm laser at a scattering angle of 90° (Yadav et al., 2018). The measurements were performed at 25 °C after diluting the samples to an appropriate concentration with ultrapure water (pH= 7.0).

### 4.8. *In vitro* release profile study

To study the *in vitro* release behavior of ROS-triggered DOX load Polymeric micelles, 1.5 mg of PEG-b-PAsp/DOX and PEG-b-PAsp-g-PBE/DOX aqueous solution was infused into a dialysis bag with 3500 DA cutoff and dialyzed against phosphate-buffered saline (PBS, 1 mM H_2_O_2_) at 37 °C on an orbital shaker in the dark. At each interval time point over a period of 48 h (1 h, 2 h, 4 h, 6 h, 10 h, 12 h, 24 h, 48 h), the concentration of DOX in the dialysate was measured by ultraviolet spectroscopy. The total volume of dialysis medium was maintained at 80 mL through the test.

### 4.9. Measurement of intracellular ROS level

Intracellular ROS generation in different cell lines was quantitatively detected using a ROS Assay Kit. Briefly, tumor cells (1 × 106 cells/mL) A549, HeLa, HepG2, MCF-7 were seeded on a coverslip in a 6-well plate and incubated overnight in a 5% CO_2_ incubator at 37 °C for attachment, respectively. DCFH-DA was added for ROS detection. The fluorescence of DCF-DA (generated after the oxidation of DCFH-DA by ROS) was measured by fluorescence analysis (Huang et al., 2018; Zhang et al., 2019).

### 4.10. Confocal laser scanning microscopy (CLSM) imaging analysis

To investigate the general intracellular distribution of free DOX, PEG-b-PAsp/DOX, PEG-b-PAsp-g-PBE/DOX, CLSM was utilized to trace the endocytosed free DOX, PEG-b-PAsp/DOX, PEG-b-PAsp-g-PBE/DOX according to previously reported procedures. 5 × 104 HeLa cells were seeded on a coverslip in 35 mm dishes and incubated overnight in a 5% CO_2_ incubator at 37 °C for attachment, respectively. Cells were first washed with phosphate-buffered saline (PBS, pH = 7.4) and treated with free DOX, PEG-b-PAsp/DOX, PEG-b-PAsp-g-PBE/DOX. After culturing for 4 h, the cells were washed with PBS three times and mixed with 500 μL 4% paraformaldehyde for 20 min. Subsequently, to further observe cell nuclei, cells were stained with DAPI (2 μg/mL) for 15 min. Cell nuclei and intracellular fluorescent free DOX, PEG-b-PAsp/DOX, PEG-b-PAsp-g-PBE/DOX were observed by CLSM (Zhao et al., 2018).

### 4.11. *In vitro* antitumor activity

The cell cytotoxicity of free DOX, PEG-b-PAsp/DOX, PEG-b-PAsp-g-PBE/DOX on HeLa cells was determined quantitatively by using MTT assay. All the cells were seeded in a 96 well plate at a density of 1 × 104 cells/well and incubated with a series of free DOX, PEG-b-PAsp/DOX, PEG-b-PAsp-g-PBE/DOX containing the same concentration ranging from 3 to 0.04 mg/L of DOX for 48 h under the same conditions.

### 4.12. Tumor model

Three- to four-week-old Balb/c female nude mice (20–22 g, Nanjing Mu Tu Medical Science and Technology Co.) were inoculated subcutaneously with 2 × 106 HeLa cells (suspended in 100 μl sterile PBS)/mouse in the right side of the flank. The tumor sizes were measured every 2 days by a digital caliper. The tumor volume was figured out according to the following equation: volume = tumor width^2^×tumor length/2. *In vivo* experiments were conducted when the tumor average volume reached 120–150 mm^3^ (Liu et al., 2016; Li et al., 2017; Wang et al., 2019).

### 4.13. Antitumor efficacy study

To evaluate the curative effects of PEG-b-PAsp-g-PBE/DOX in vivo, nude mice bearing HeLa tumor were administrated twice a week with PBS, free DOX·HCl, PEG-b-PAsp-g-PBE, PEG-b-PAsp/DOX, PEG-b-PAsp-g-PBE/DOX (containing same concentration of DOX) nanoparticles, respectively. Eighteen days later, treated mice were sacrificed, and the tumor tissues were removed from the bodies for measurement. Tumors and major organs (heart, liver, spleen, lung and kidney) were dissected from mice and hematoxylin–eosin (H&E) staining.

## Supplementary Material

Supplemental Material
