# Supplementary material for: Phenylboronic ester-modified anionic micelles for ROS-stimuli response in HeLa cell
Source: Drug Deliv. 2020 May 12;27(1):681–90. doi: 10.1080/10717544.2020.1748761 (PMC7269054; doi:10.1080/10717544.2020.1748761)
Supplement: Supplemental Material [file IDRD_A_1748761_SM5572.docx]

**Supporting Information**

Phenylboronic Ester-Modified Anionic Micelles for ROS-[Stimuli Response](javascript:;) in HeLa Cell

*Qi Y. Wang^1⊥^, Yi S. Xu^1⊥^, Nan X. Zhang^1^, Zhi P. Dong^1^, Bo N. Zhao^1^, Lin C. Liu^2^, Tao Lu*^1^, Yue Wang***^1^*

^1^ Key Laboratory of Biomedical Functional Materials, School of Sciences, China Pharmaceutical University, Nanjing 211198, Jiangsu Province, China.

2 Department of Rheumatology, Zhongda Hospital, School of Medicine, Southeast University, Nanjing, Jiangsu, China.

**
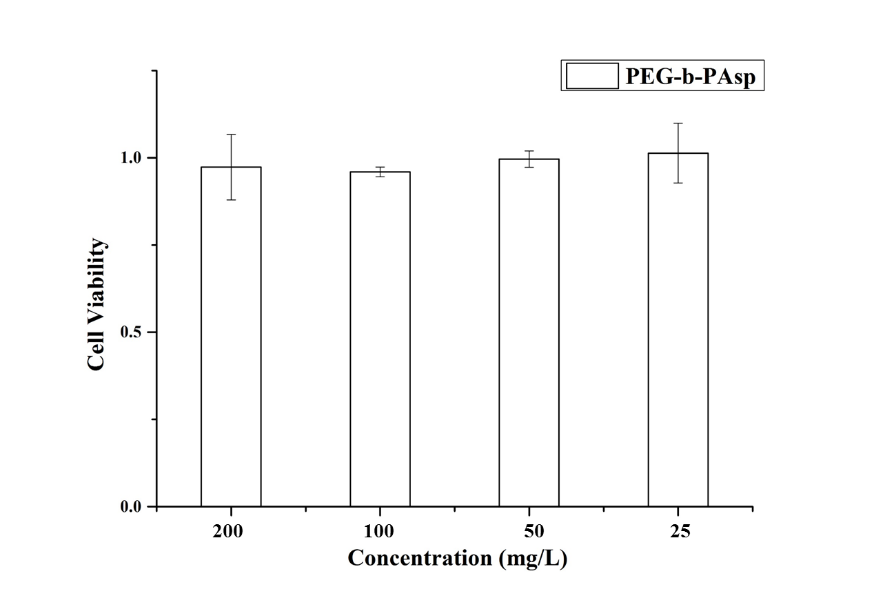
**

**Figure S1.** Cell inhibition of PEG-b-PAsp on L-O2 at different concentrations

**
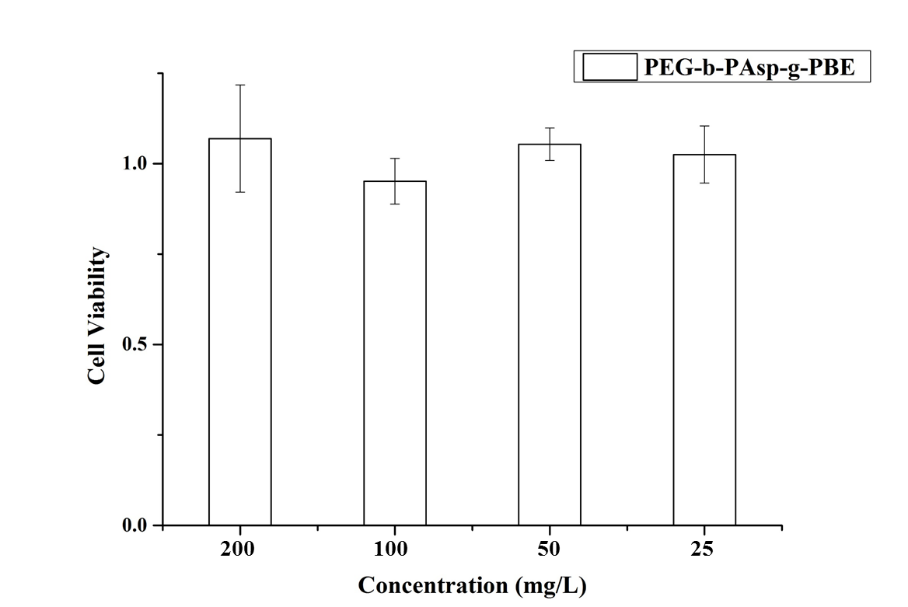
**

**Figure S2.** Cell inhibition of PEG-b-PAsp-g-PBE on L-O2 cells at different concentrations


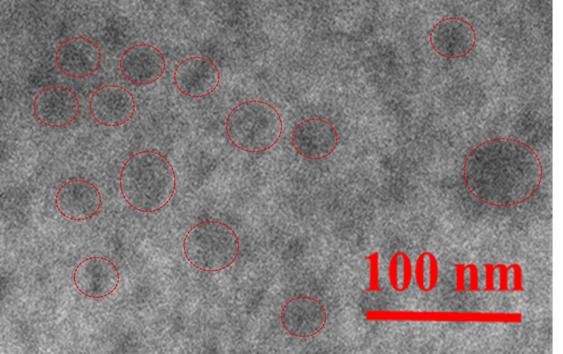


**Figure S3**.TEM image of the PEG_45_-b-PAsp_72_-g-PBE_41_**
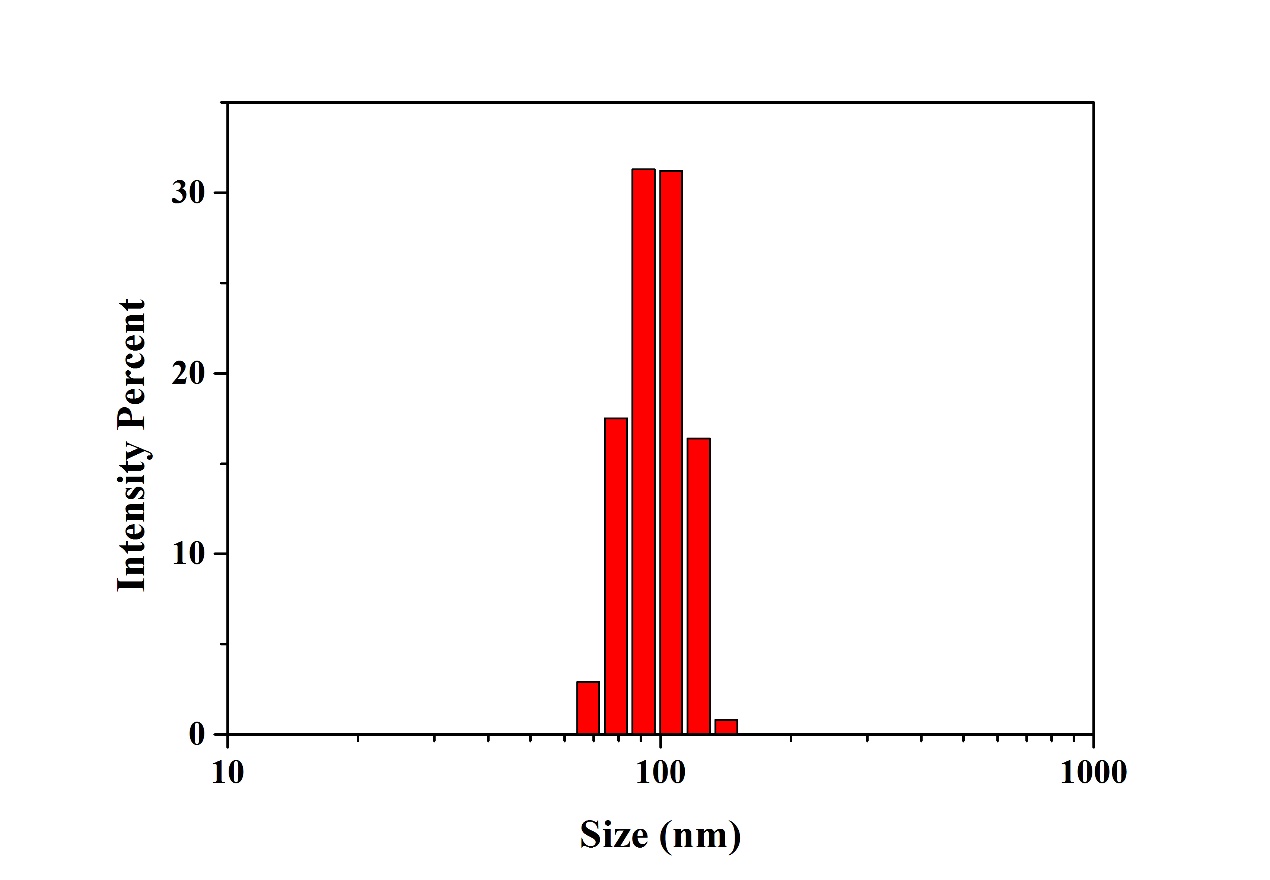
**

**Figure S4.** Size distribution of the PEG_45_-b-PAsp_72_ in PBS 7.4


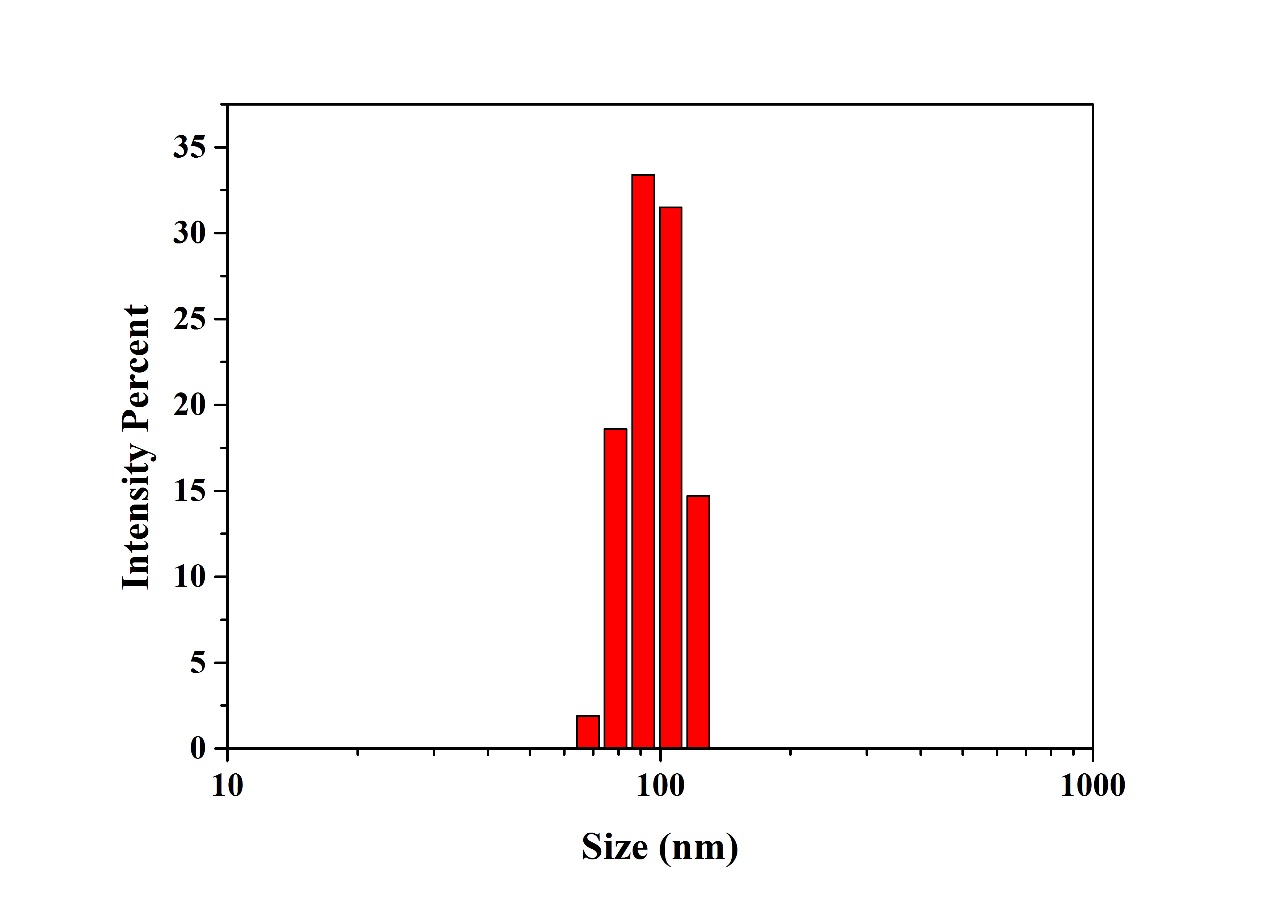


**Figure S5.** Size distribution of the PEG_45_-b-PAsp_72_-g-PBE_41_ in PBS 7.4


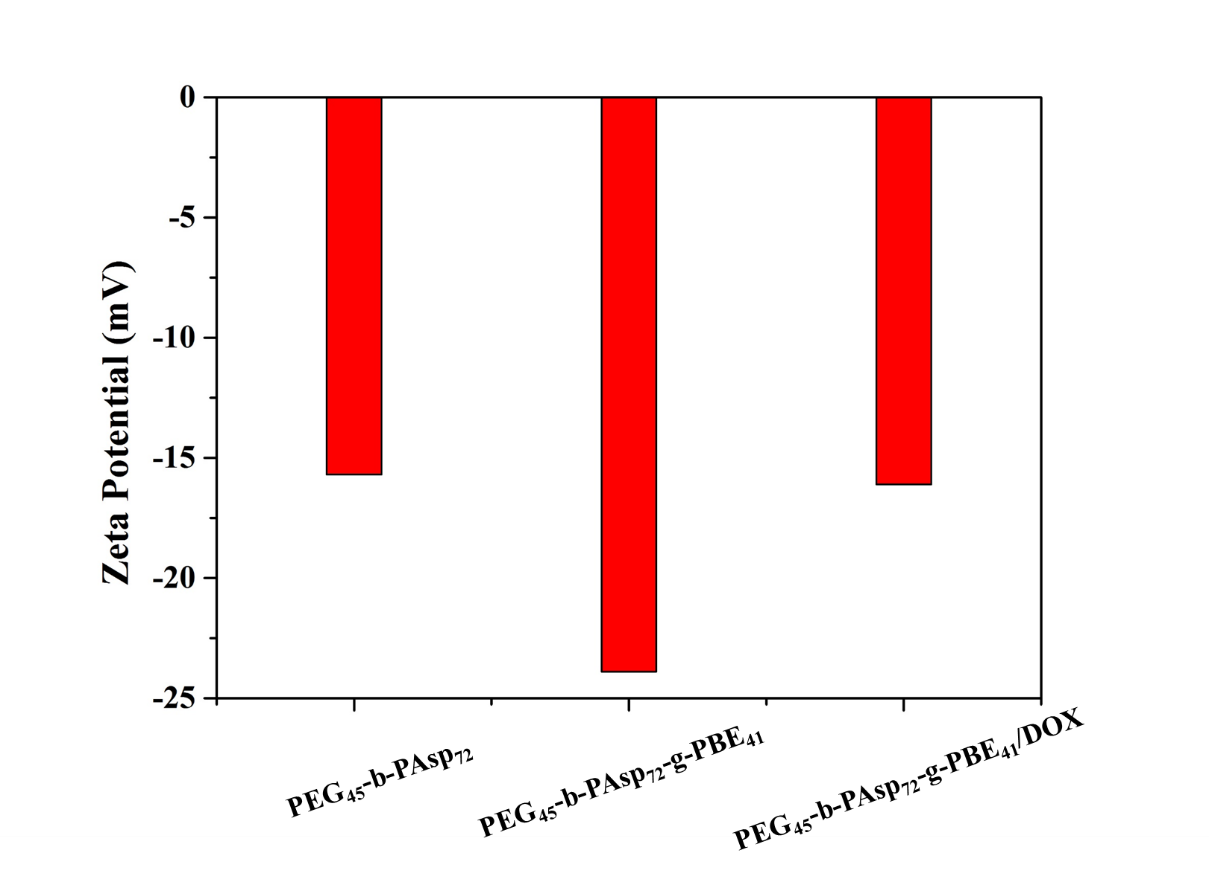


**Figure S6.** Zeta potential of polymeric micelles


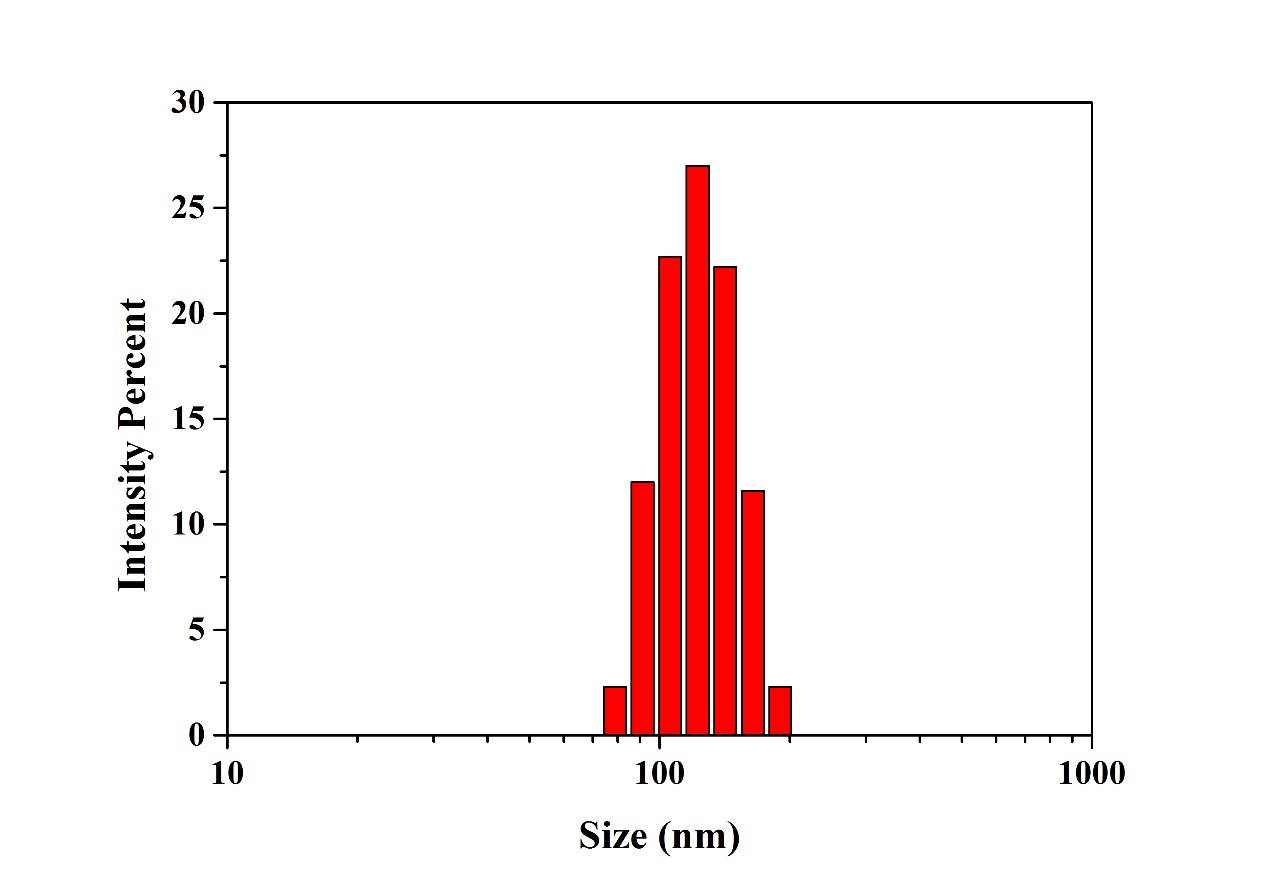


**Figure S7.** Size distribution of the PEG_45_-b-PAsp_72_-g-PBE_41_/DOX in PBS 7.4
